# The Type-Specific Neutralizing Antibody Response Elicited by a Dengue Vaccine Candidate Is Focused on Two Amino Acids of the Envelope Protein

**DOI:** 10.1371/journal.ppat.1003761

**Published:** 2013-12-05

**Authors:** Laura A. VanBlargan, Swati Mukherjee, Kimberly A. Dowd, Anna P. Durbin, Stephen S. Whitehead, Theodore C. Pierson

**Affiliations:** 1 Viral Pathogenesis Section, Laboratory of Viral Diseases, National Institute of Allergy and Infectious Diseases, National Institutes of Health, Bethesda, Maryland, United States of America; 2 Johns Hopkins School of Public Health, Baltimore, Maryland, United States of America; 3 Laboratory of Infectious Diseases, National Institute of Allergy and Infectious Diseases, National Institutes of Health, Bethesda, Maryland, United States of America; Institut Pasteur, France

## Abstract

Dengue viruses are mosquito-borne flaviviruses that circulate in nature as four distinct serotypes (DENV1-4). These emerging pathogens are responsible for more than 100 million human infections annually. Severe clinical manifestations of disease are predominantly associated with a secondary infection by a heterotypic DENV serotype. The increased risk of severe disease in DENV-sensitized populations significantly complicates vaccine development, as a vaccine must simultaneously confer protection against all four DENV serotypes. Eliciting a protective tetravalent neutralizing antibody response is a major goal of ongoing vaccine development efforts. However, a recent large clinical trial of a candidate live-attenuated DENV vaccine revealed low protective efficacy despite eliciting a neutralizing antibody response, highlighting the need for a better understanding of the humoral immune response against dengue infection. In this study, we sought to identify epitopes recognized by serotype-specific neutralizing antibodies elicited by monovalent DENV1 vaccination. We constructed a panel of over 50 DENV1 structural gene variants containing substitutions at surface-accessible residues of the envelope (E) protein to match the corresponding DENV2 sequence. Amino acids that contribute to recognition by serotype-specific neutralizing antibodies were identified as DENV mutants with reduced sensitivity to neutralization by DENV1 immune sera, but not cross-reactive neutralizing antibodies elicited by DENV2 vaccination. We identified two mutations (E126K and E157K) that contribute significantly to type-specific recognition by polyclonal DENV1 immune sera. Longitudinal and cross-sectional analysis of sera from 24 participants of a phase I clinical study revealed a markedly reduced capacity to neutralize a E126K/E157K DENV1 variant. Sera from 77% of subjects recognized the E126K/E157K DENV1 variant and DENV2 equivalently (<3-fold difference). These data indicate the type-specific component of the DENV1 neutralizing antibody response to vaccination is strikingly focused on just two amino acids of the E protein. This study provides an important step towards deconvoluting the functional complexity of DENV serology following vaccination.

## Author Summary

Despite decades of research, there remains a critical need for a dengue virus (DENV) vaccine. Vaccine development efforts are complicated by a requirement to protect against four DENV serotypes (DENV1-4), and incomplete immunity as a risk factor for severe disease. Antibodies play a major protective role against DENV. However, they also have been implicated in severe clinical manifestations of DENV infection. The antibody response to DENV is composed of antibodies that neutralize only the infecting DENV serotype (type-specific), as well as those that are cross-reactive. Cross-reactive antibodies are hypothesized to contribute to severe dengue following heterologous infections. Identifying DENV epitopes that are targets of type-specific neutralizing antibodies may facilitate vaccine development and the identification of correlates of protection. In this study, we identified amino acids on DENV1 recognized by type-specific neutralizing antibodies elicited by DENV1 vaccination. Our results indicate that the type-specific DENV1 response is remarkably focused on just two regions of the DENV1 envelope protein. Furthermore, a significant contribution of antibodies with this specificity was a common feature among vaccine recipients. This study identifies targets of neutralizing antibodies elicited by DENV1 vaccination and provides an important first step toward identifying epitopes recognized by each component of a tetravalent vaccine.

## Introduction

Dengue virus (DENV) is a mosquito-transmitted flavivirus responsible for 390 million human infections each year [1]. Four related serotypes (DENV1-4) circulate in virtually all tropical and sub-tropical regions of the world [2]. While DENV infection is often subclinical, clinical symptoms of dengue fever (DF) include a self-limiting febrile illness, myalgia, rash, and retro-orbital pain [3]. A more severe clinical illness (dengue shock syndrome/dengue hemorrhagic fever) involving capillary leakage, thrombocytopenia, and hemorrhage has been associated with secondary infections by a heterologous DENV serotype and higher viral loads *in vivo* [4], [5]. The incidence of severe DENV disease is rising globally due to increasing co-circulation of multiple DENV serotypes in endemic areas [2], [6]. Currently, there are no specific treatments or approved vaccines for DENV infection.

Flaviviruses encapsidate a single-stranded RNA genome of positive-sense polarity. This ∼11 kb genomic RNA is translated as a single open reading frame that is cleaved in infected cells by cellular and viral proteases into at least ten proteins [7]. The virus encodes three structural proteins (envelope (E), premembrane (prM), and capsid (C)) that associate with a lipid envelope and the viral genome to form the virion [8]. Flavivirus assembly occurs on virus-induced membranes derived from the endoplasmic reticulum (ER) [9]–[13], resulting in the budding of non-infectious immature virus particles into the lumen. The E protein of immature virions exists as heterotrimeric spikes in complex with the prM protein; sixty of these spikes are organized on the virion with icosahedral symmetry [14]–[16]. During egress through the secretory pathway, prM is cleaved by a cellular furin-like protease to generate the mature infectious virus particle [17]–[19]. Mature virions are characterized by a dense array of antiparallel E protein dimers orientated roughly parallel to the surface of the virion [20]–[22]. In many cases, this arrangement of E proteins imposes steric constraints for epitope recognition by antibodies [23], [24]. The virion maturation process is inefficient for many mosquito-borne flaviviruses, including DENV. Partially mature viruses with structural features of both mature and immature particles may be infectious and differentially interact with antibodies as a function of their prM content (reviewed in [25]).

**Figure 1:**
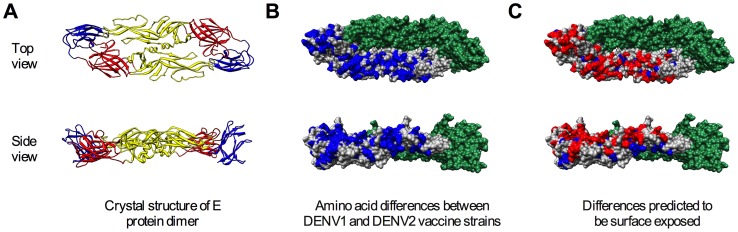
Surface-accessible residues that differ between DENV1 and DENV2 identified for mutagenesis. The flavivirus E protein contains three distinct domains (DI–III) and forms antiparallel dimers on the mature virus particle. Shown is the crystal structure of the soluble ectodomain of the DENV2 E protein dimer (PDB 1OAN) as viewed from the top (top panels) and side (bottom panels). (**A**) Ribbon diagram of the E protein with DI, II, and III colored in red, yellow, and blue, respectively [102]. (**B**) The 130 amino acid residues in the soluble ectodomain that differ between the DENV1 and DENV2 components of the NIAID candidate tetravalent vaccine are highlighted in blue on one E protein; the second E protein of the dimer is shaded in green. (**C**) Surface accessibility was estimated using solvent accessible surface areas of the residues determined from the crystal structure (UCSF Chimera package), with a cut-off value of 30 Å^2^ [103]. Residues selected for study were restricted to the top of the dimer. The 68 residues identified as surface-accessible differences between DENV1 WP and DENV2 NGC are shown in red.

The humoral response plays an important role in protection against flaviviruses (reviewed in [26]). Development of a neutralizing antibody response is an established correlate of protection following vaccination against yellow fever virus (YFV), Japanese encephalitis virus (JEV), and tick-born encephalitis virus (TBEV) [27]–[30]. Passive transfer of monoclonal antibodies (mAbs) is protective in several animal models of flavivirus infection, including DENV [31]–[36]. The flavivirus E protein (**Figure 1a**) is the principal target of neutralizing antibodies [37]. Studies with murine and human mAbs have identified neutralizing epitopes on all three structural domains of E (DI–III) [23], [24], [35], [36], [38]–[47]. Recent studies with human mAbs suggest the antibody repertoire may differ from that observed in mice [39], [48]–[52], and have identified a quaternary epitope composed of surfaces on two adjacent E proteins [46], [48], [52]. Antibodies that bind prM also have been identified frequently in studies of human mAbs and typically possess limited neutralization potential *in vitro* [39], [49], [51], [53]. Beyond a capacity to directly neutralize the infectivity of virions, antibodies may protect the host via effector functions orchestrated by the constant region of the antibody molecule [54]–[57].

Development of a DENV vaccine has been a focus of considerable effort for decades. While the dramatic success of other flavivirus vaccines [28], [58], [59] and the pioneering work of Sabin and colleagues [60] suggest effective DENV vaccination is possible, several unique challenges exist. A DENV vaccine must simultaneously protect against four different viruses. In addition, vaccination must not sensitize the recipient to more severe manifestations of disease in the event of breakthrough. The antibody response to DENV results in the production of both type-specific (TS) and cross-reactive (CR) antibodies that may vary significantly with respect to their capacity to neutralize virus infection [35], [36], [38], [61]–[63]. CR antibodies are hypothesized to contribute to severe clinical outcomes of DENV infection via a process called antibody-dependent enhancement of infection [64]. The extensive cross-reactivity of the DENV antibody response complicates serological studies of these viruses and the identification of immune correlates of protection [65], [66]. For example, a recent large phase IIb trial of a live-attenuated tetravalent DENV vaccine revealed modest protective efficacy (∼30% overall) with no protection at all observed for DENV2 [67]. Importantly, despite the absence of protection, vaccine-induced neutralizing antibody was observed for DENV2. Whether this increase in neutralizing titer was associated with a TS- or CR-response is unknown. This trial underscores the importance of understanding the functional complexity of the DENV antibody response.

In this study we sought to identify the immunodominant epitopes recognized by TS-neutralizing antibodies elicited by DENV vaccination of humans. We constructed libraries of DENV1 variants containing substitutions in the E protein at surface exposed residues that differ between the DENV1 and DENV2 components of the NIAID tetravalent vaccine candidate [68]. We then screened this library for a reduction in sensitivity to neutralization by sera from DENV1 vaccine recipients, but not DENV2 immune sera from vaccinated subjects. Remarkably, these studies identified two amino acids (E126 and E157) that when mutated significantly reduced the DENV1 TS-neutralizing response of more than 77% of recipients of a monovalent DENV1 vaccine. Our studies, for the first time, identify functionally significant epitopes that comprise a TS-neutralizing response and provide insight into the complexity of the DENV humoral response.

## Results

### Strategy for identifying epitopes recognized by type-specific antibodies elicited by DENV1 vaccination

The goal of this study was to identify epitopes recognized by DENV1 TS-neutralizing antibodies elicited by vaccination. Our analyses employed immune sera collected during the clinical evaluation of individual components of the NIAID tetravalent vaccine candidate (reviewed by [68]). The attenuated DENV1 in this vaccine (rDEN1Δ30) is derived from the South Pacific genotype 4 Western Pacific (WP) strain [69]. The DENV2 component of the tetravalent formulation (rDEN2/4Δ30(ME)) is the Southeast Asian genotype 2 New Guinea C strain (NGC) [70]. The envelope proteins of these viruses differ by 158 amino acids (**Figure 1b**); 68 amino acids that differ between these two strains are predicted to be accessible to solvent on the surface of the mature DENV virion (**Figure 1c**, see Materials and Methods) [71]. Both rDEN1Δ30 and rDEN2/4Δ30(ME) vaccines were tested as monovalent formulations in phase I clinical studies in humans [72], [73]. To identify amino acids recognized by TS-neutralizing antibodies, we created a panel of DENV1 variants to replace surface-accessible residues on the mature DENV1 virion with those found on DENV2 NGC (detailed below). This panel of mutants was then screened using pooled immune sera collected during monovalent DENV1 and DENV2 vaccine studies for those exhibiting reduced sensitivity to neutralization by DENV1, but not DENV2, immune sera.

Construction and functional characterization of libraries of DENV1 E protein variants were performed using DENV reporter virus particles (RVPs). RVPs are produced by complementation of a self-replicating sub-genomic flavivirus replicon with a plasmid encoding viral structural genes. These virus particles are capable of only a single round of infection and allow virus entry to be scored as a function of reporter gene expression. Flavivirus RVPs have been used extensively to study the functional properties of anti-flavivirus antibodies [33], [74]–[78]. This technology allows for the rapid generation of structural gene variants by exchanging the plasmids used in complementation studies, simplifying the construction and characterization of a large library of DENV1 mutants. Furthermore, because the RVPs are not passaged in cell culture, the genetic stability of a particular mutation does not limit its utility in neutralization studies.

**Figure 2:**
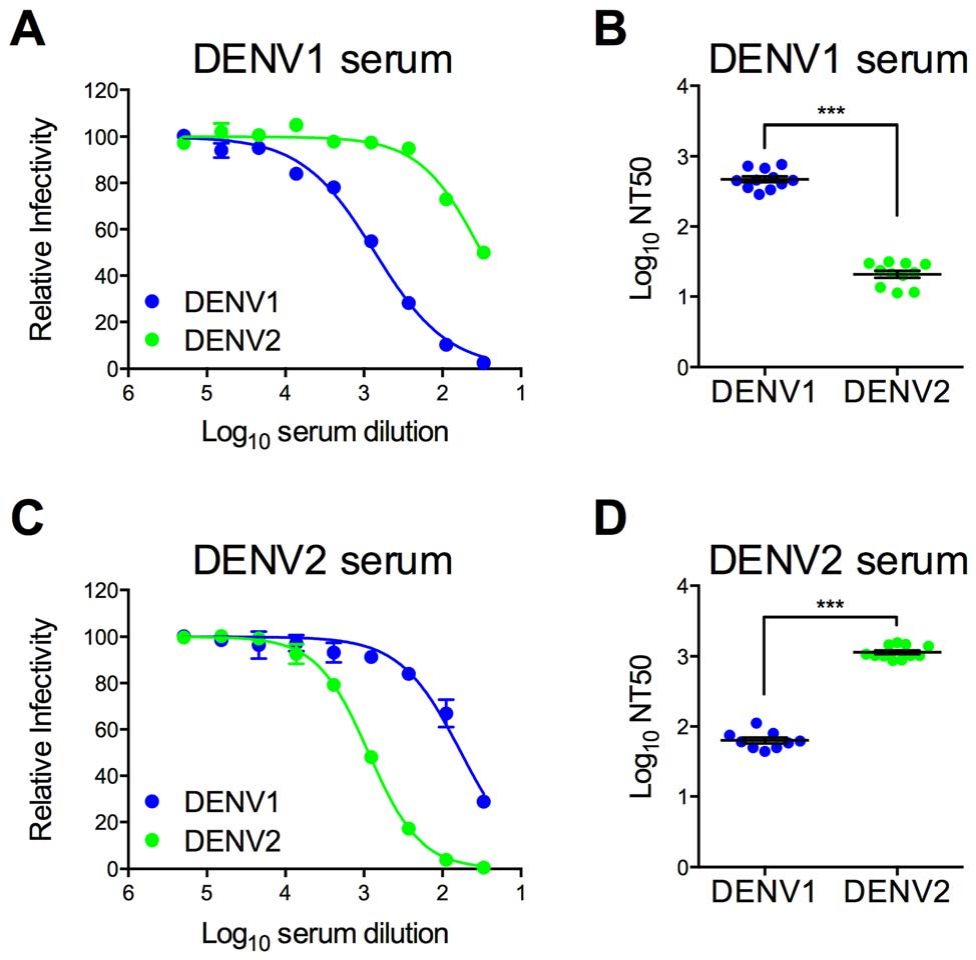
Type-specific neutralization by DENV immune sera from a clinical vaccine trial. Pooled immune sera from a DENV1 or DENV2 vaccine study were tested for their capacity to neutralize DENV1 and DENV2 RVPs. Sera pooled from multiple vaccinees (two and three for DENV1 and DENV2, respectively) were used in neutralization experiments by incubating RVPs with serial dilutions of immune sera for one hour at room temperature, before addition to Raji-DCSIGNR cells. After incubation at 37°C for two days, infection was measured using flow cytometry. Dose response curves for (**A**) DENV1 sera and (**C**) DENV2 sera are expressed relative to the infectivity of the RVPs in the absence of serum. The concentration of sera indicated on the x-axis is expressed as Log_10_ (dilution factor of serum). Error bars represent the standard error of duplicate infections. Dose response curves shown in (**A**) and (**C**) are representative of 11 and nine independent experiments, respectively, performed using at least three independent RVP preparations. Neutralization titer (NT50) values were determined by nonlinear regression analysis using Prism software (GraphPad), and are summarized for (**B**) DENV1 sera and (**D**) DENV2 sera. Error bars represent standard error of the mean. ***p<0.0001.

Immune sera used to characterize and screen DENV1 mutant libraries were pooled from recipients of DENV1 or DENV2 vaccine candidates (two and three vaccine recipients, respectively) [72], [73]. The neutralization titer (NT50) of antibodies in these pooled serum samples was determined using wild type (WT) DENV1 WP and DENV2 NGC RVPs. As expected, antibodies present in the DENV1 immune sera efficiently neutralized DENV1 RVPs (Log NT50 2.67±0.04, n = 11) (**Figure 2a and b**). Cross-reactive neutralizing antibodies in the sera, measured using DENV2 RVPs (Log NT50 1.32±0.05, n = 11), were significantly less potent when compared to DENV1 neutralization (23-fold; p<0.0001). A similar pattern of TS- and CR- neutralization was observed in reciprocal studies with DENV2 immune sera (**Figure 2c and d**), which neutralized DENV2 RVPs (Log NT50 3.06±0.03, n = 11) at significantly greater dilutions than DENV1 RVPs (Log NT50 1.80±0.04, n = 9) (20-fold, p<0.0001).

### Construction of a library of DENV1 variants with individual substitutions to match DENV2 at surface-accessible differences

**Figure 3:**
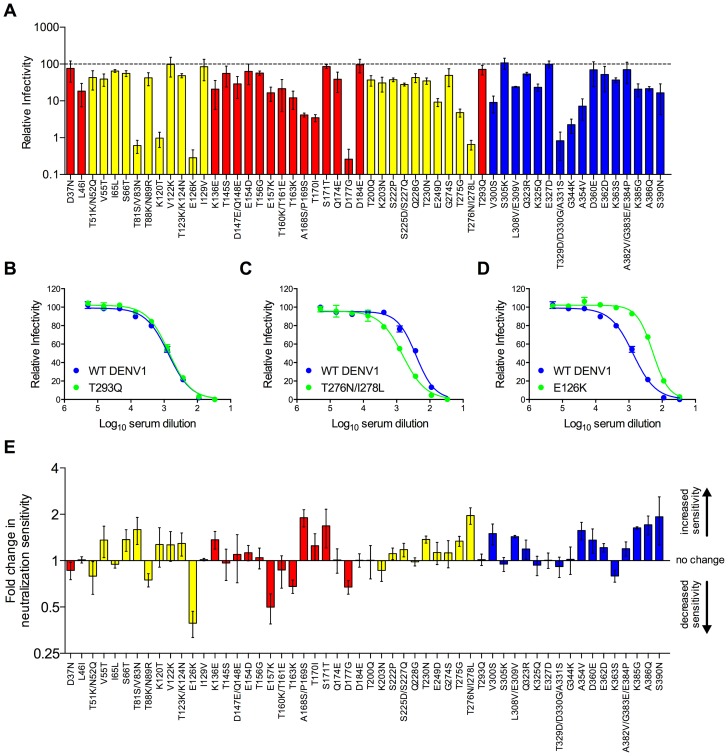
Impact of mutations on the neutralization potency of DENV1 immune serum. A panel of 54 DENV1 RVP variants containing single, double, or triple amino acid changes was constructed by site-directed mutagenesis. This panel represented all surface-accessible residues identified in **Figure 1c**. (**A**) The infectious RVP titer for each variant was determined concurrently with WT DENV1 using Raji-DCSIGNR cells. Values are the mean relative titer as compared to WT DENV1 infectivity measured in parallel from at least two independent RVP preparations; error bars represent standard error of the mean. Variants located in domains I, II and III of the E protein are colored in red, yellow, and blue, respectively. The sensitivity of the 54 DENV1 variants to neutralization by DENV1 immune serum was compared to WT DENV1 as described in **Figure 2**. (**B–D**) Examples of the three patterns of neutralization by DENV1 immune sera observed are shown. Error bars represent the standard error of duplicate infections. (**E**) Neutralization sensitivities of all DENV1 variants to DENV1 immune sera are depicted as the mean fold increase in neutralization sensitivity ([NT50 variant]/[NT50 WT]); error bars represent standard error of the mean of 2–5 independent experiments.

**Figure 4:**
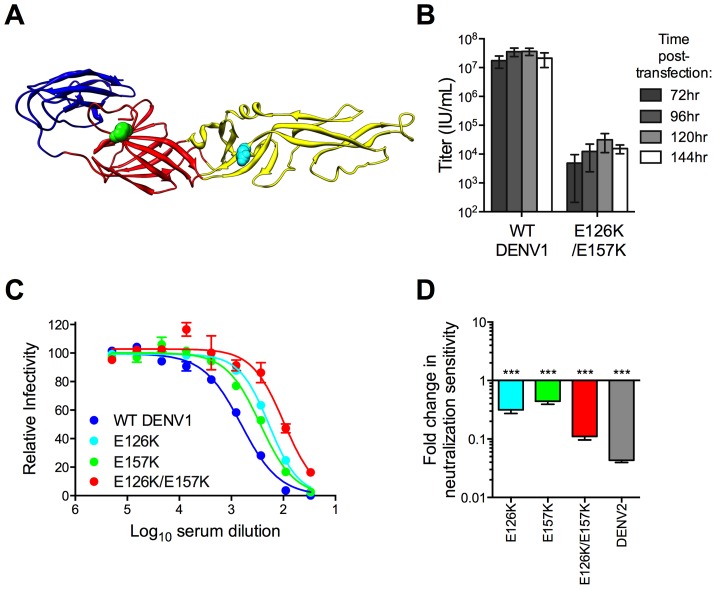
Combined effect of DENV1 mutations E126K/E157K on the neutralization potency of DENV1 immune serum. (**A**) The location of residues E126 and E157 are highlighted on the E protein crystal structure as cyan and green spheres, respectively. E protein domains are colored as in **Figure 1**. (**B**) Infectious titer of DENV1 E126K/E157K RVPs harvested at four time points post-transfection was determined in parallel studies with WT DENV1 using Raji-DCSIGNR cells; error bars represent the standard error of the mean of 2–4 independent experiments. (**C and D**) DENV1 E126K/E157K RVPs were tested for sensitivity to neutralization by the DENV1 immune serum. (**C**) Representative dose-response curves for the single and double mutants are shown; error bars represent the standard error of duplicate infections. (**D**) The neutralization sensitivity is summarized as the fold-increase in NT50 from WT DENV1 for the single mutants E126K and E157K (n = 10), the E126K/E157K double mutant (n = 11), and DENV2 (n = 11); error bars represent the standard error of the mean. ***p<0.0001 for a comparison of the Log NT50 values to WT DENV1 by an ANOVA followed by Tukey's multiple comparisons test.

To disrupt TS epitopes recognized by DENV1 immune sera, we created a library of RVPs in which the 68 surface-accessible residues that vary between the DENV1 and DENV2 components of the vaccine (**Figure 1**) were used to guide construction of a panel of 54 DENV1 variants containing only one, two, or three substitutions each; in cases where adjacent residues were selected for mutagenesis they were introduced into the same RVP. All 54 DENV1 variants were infectious, albeit to differing degrees (n = 2–5, **Figure 3a**). The sensitivity of each variant in this panel to neutralization by DENV1 immune sera was tested, and three patterns emerged. The majority of DENV1 variants (81%) were neutralized to the same extent as WT DENV1 tested in pairwise experiments (**Figure 3b and e**). Eight variants in this panel (15%) revealed a small (1.5- to 2-fold) but reproducible increase in sensitivity to neutralization (**Figure 3c and e**). These variants were also typically more sensitive to neutralization by the DII-fusion loop reactive mAb E60 (**Figure S1**). Mechanisms with the potential to increase neutralization sensitivity in this context are discussed below. Finally, two mutants, E126K and E157K, exhibited a statistically significant decrease in sensitivity to neutralization by pooled DENV1 sera (3.5-fold (n = 10, p<0.0001) and 2.4-fold (n = 10, p<0.0001), respectively) (**Figure 3d and e** **,** **Figure 4c and d**). Neither of these two mutations conferred an altered sensitivity to neutralization by mAb E60 (**Figure S1**).

To investigate whether a single DENV1 variant encoding both E126K and E157K mutations had an even further reduction in neutralization sensitivity, we combined them into a single construct (**Figure 4a**). RVPs produced using this DENV1 E126K/E157K variant were able to infect cells, albeit with greatly reduced titer as compared to WT DENV1 RVPs (>1,000 fold, p<0.001 for all time points, n = 2–4) (**Figure 4b**). Neutralization studies using single and double mutants revealed the DENV1 E126K/E157K variant was more resistant to neutralization by DENV1 immune sera than either single mutant (Log NT50 1.79±0.06, n = 11) (p<0.0001 for both comparisons) (**Figure 4c and d**).

### Characterization of the E126K/E157K DENV1 variant

Our screening data with pooled immune sera suggested antibodies that bound epitopes containing residues E126 and E157 were a functionally significant component of the polyclonal antibody response to DENV1 vaccination. To validate this interpretation, we performed control experiments to address aspects of flavivirus biology with the potential to reduce the apparent sensitivity of flaviviruses to antibody-mediated neutralization.

**Figure 5:**
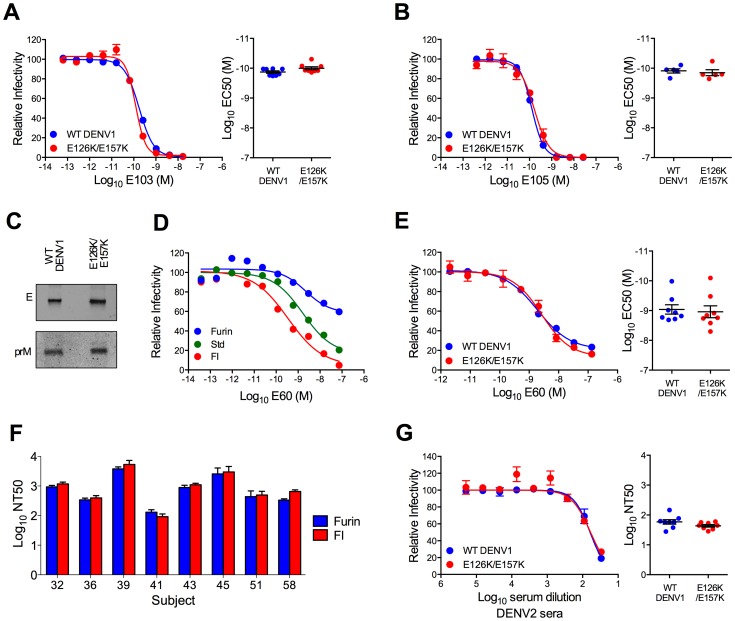
Characterization of the E126K/E157K DENV1 variant. (**A** and **B**) WT DENV1 and DENV1 E126K/E157K RVPs were evaluated for sensitivity to neutralization by DENV1 DIII-binding mAbs. Within each panel, representative dose response curves for each antibody are shown on the left; error bars represent the standard error of duplicate infections. Plots on the right show the EC50 values obtained from independent experiments; error bars represent standard error of the mean. The antibodies tested were (**A**) mAb E103 (n = 8, p = 0.14), and (**B**) mAb E105 (n = 5, p = 0.57). (**C**) WT DENV1 and E126K/E157K RVPs were analyzed by Western blot with an anti-E mAb and an anti-prM mAb. The efficiency of prM cleavage was evaluated on blots normalized by loading equivalent E protein. (**D**) WT DENV1 RVPs were produced using standard methods (Std), in the presence of high levels of human furin expression (furin), or in cells treated with furin inhibitor (FI) and then tested for sensitivity to neutralization by mAb E60. Three independent experiments were performed; representative dose response curves are shown. Error bars represent the standard error of duplicate infections. (**E**) DENV1 E126K/E157K was evaluated for sensitivity to neutralization by mAb E60 as compared to WT DENV1. Representative dose response curves are shown on the left; error bars represent the standard error of duplicate infections. EC50 values from independent experiments are shown in the right panel; error bars represent standard error of the mean (n = 8, p = 0.68). (**F**) Furin- and FI-DENV1 RVPs were tested for sensitivity to neutralization by sera from DENV1 vaccine recipients. Error bars represent standard error from three independent experiments. Statistical evaluation using ANOVA followed by a Šidák correction for multiple comparisons failed to identify a difference between Furin- and FI-DENV1 RVPs (p>0.05 for each pair). (**G**) DENV1 E126K/E157K was evaluated for sensitivity to neutralization by pooled sera from DENV2 vaccine recipients. A representative dose response curve is shown on the left; error bars represent the standard error of duplicate infections. NT50 values obtained from eight independent experiments are shown on the right; error bars represent standard error of the mean (p = 0.08).

First, we conducted experiments to demonstrate that the reduced sensitivity of the E126K/E157K variant to neutralization was not an artifact of its low titer and/or an overall disruption of the antigenic surface of the virion. An assumption of quantitative neutralization studies is that the observed neutralizing activity is dependent solely on the concentration of antibody and its affinity/avidity for viral antigens; the results of neutralization studies should be independent of the amount of antigen/virus in the experiment [79], [80]. Excess antigen in virus preparations (due to a reduced specific infectivity) may shift antibody dose-response curves towards higher concentrations of antibody if present in sufficient amounts to reduce the concentration of free antibody in solution [77]. To rule out the confounding effects of excess antigen in preparations of the E126K/E157K variant, we performed control studies using TS-neutralizing mAbs that have been mapped to the DIII lateral ridge [35]. Neutralization by these mAbs should not be affected by the DI/DII mutations. Both mAb DENV1-E103 (**Figure 5a**, n = 8) and mAb DENV1-E105 (**Figure 5b**, n = 5) neutralized WT DENV1 and DENV1 E126K/E157K RVPs to an equivalent extent (p = 0.14 and 0.57, respectively). Confirmatory studies with six additional DENV1 mAbs yielded similar results; in each instance we observed less than a two-fold difference in the EC50 of WT and the E126K/E157K variant (**Figure S2**, n = 2). Altogether, these results demonstrate that studies of the neutralization sensitivity of the DENV1 E126K/E157K variant are not confounded by excess antigen arising from a reduction in the specific infectivity of the virus.

We have shown previously that changes in the efficiency of virion maturation can markedly impact the sensitivity of flaviviruses to neutralization by antibodies [74]. Because mutations in the E protein have the potential to impact the efficiency of prM cleavage, we investigated the maturation state of the DENV1 E126K/E157K variant using biochemical and functional approaches. The prM content of the DENV1 E126K/E157K variant was found to be similar to WT by Western blot (**Figure 5c**). The DII-FL-reactive mAb E60 neutralizes flaviviruses in a maturation state-dependent fashion and serves as a sensitive functional probe for changes in virion structure that expose this otherwise cryptic epitope on the mature virion [74], [81]. Manipulating the efficiency of prM cleavage modulates the potency of E60 against DENV1 WP; increasing the efficiency of virion maturation reduces sensitivity of the virion to neutralization (**Figure 5d**). That no significant difference in the potency of E60 was observed when studying WT DENV1 (EC50 = 1.3×10^−9^±2×10^−10^ M, n = 8) and DENV1 E126K/E157K (EC50 = 1.9×10^−9^±5×10^−10^ M, n = 8) (p = 0.68) (**Figure 5e**) suggests the efficiency of virion maturation of WT and the E126K/E157K variant are similar on infectious viruses. Finally, using RVP populations in which the efficiency of prM cleavage was greatly enhanced (furin RVPs) or reduced via treatment of cells with furin inhibitor (FI RVPs) and DENV1 immune sera from eight vaccine recipients, we established that the TS-neutralizing activity in these sera was not markedly sensitive to the maturation state of the virus particle (**Figure 5f**). In each instance, furin- and FI-RVPs were neutralized equivalently. Altogether our data strongly suggest that the reduction in sensitivity of the DENV1 E126K/E157K variant to neutralization by TS antibodies present in vaccine immune sera is not an artifact of a change in the efficiency of virion maturation.

The accessibility of antibody epitopes on the virion may also be modulated by changes in the structure of the virion as it samples multiple conformations at equilibrium (known as viral “breathing”) [81]–[85]. The impact of viral dynamics on antibody-mediated neutralization is most readily observed with antibodies that bind epitopes predicted to be poorly exposed on the surface of the mature virion. The neutralization potency of E60 is also sensitive to viral structural dynamics [81]. That E60 neutralized both WT and the DENV1 E126K/E157K variant with equivalent efficiency (**Figure 5e**) indicates that the reduced sensitivity of this variant to neutralization by DENV1 immune sera is not a result of a change in the extent of viral “breathing”.

Finally, studies with pooled DENV2 immune sera revealed these mutations only altered sensitivity to TS antibodies and not CR antibodies. The sensitivity of DENV1 E126K/E157K RVPs to neutralization by cross-reactive DENV2 immune sera was similar to WT DENV1 RVPs (1.3-fold, n = 8, p = 0.08) (**Figure 5g**). Together, these experiments support the conclusion that mutation at residues E126K and E157K specifically reduces neutralization by TS antibodies elicited by DENV1 vaccination. Furthermore, the reduced sensitivity of variant E126K/E157K to immune sera from DENV1, but not DENV2, vaccinated individuals was not an artifact of changes in the structural dynamics of the virion, the efficiency of virion maturation, gross changes in the antigenic structure of the virion, or antigen excess in the neutralization assay.

### Prevalence of an epitope recognized by TS antibodies present in the immune sera of DENV1-vaccinated subjects

**Figure 6:**
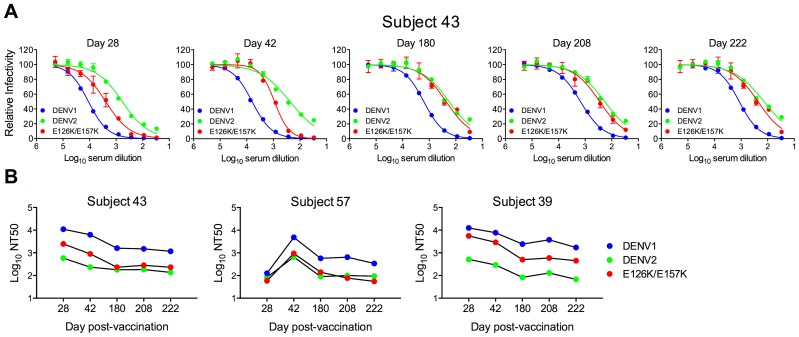
Longitudinal analysis of the effects of DENV1 E126K/E157K mutations on serum neutralizing activity. Sera collected from three DENV1 vaccine recipients at five times post-vaccination were tested for a capacity to neutralize WT DENV1, DENV2, and DENV1 E126K/E157K RVPs. (**A**) Dose-response curves for immune sera from one subject are shown. Error bars represent the standard error of duplicate infections. (**B**) NT50 values for each curve were determined by nonlinear regression analysis using Prism software (GraphPad), and are summarized for the three subjects.

To determine the prevalence of neutralizing antibodies that target epitopes incorporating residues E126 and E157, we next performed longitudinal and cross-sectional studies of immune sera obtained from individual recipients of a DENV1 live-attenuated vaccine candidate [86]. Participants in this vaccine study were administered vaccine on days 0 and 180, as described previously [86]. Neutralization studies with sera from three subjects collected at days 28, 42, 180, 208, and 222 post-vaccination were performed using WT DENV1, WT DENV2, and DENV1 E126K/E157K RVPs. These three subjects were selected for study because they represented both high and low neutralizing antibody responses among participants in the phase I study. Dose response curves and summary NT50 values of sera from longitudinally tested subjects are shown in **Figure 6**. As anticipated, sera from post-vaccination samples neutralized DENV1 more efficiently than DENV2. In each instance, neutralization activity declined over time and was not boosted significantly by a second dose of vaccine, consistent with previous reports (**Figures 6** **and** **7**) [86].

**Figure 7:**
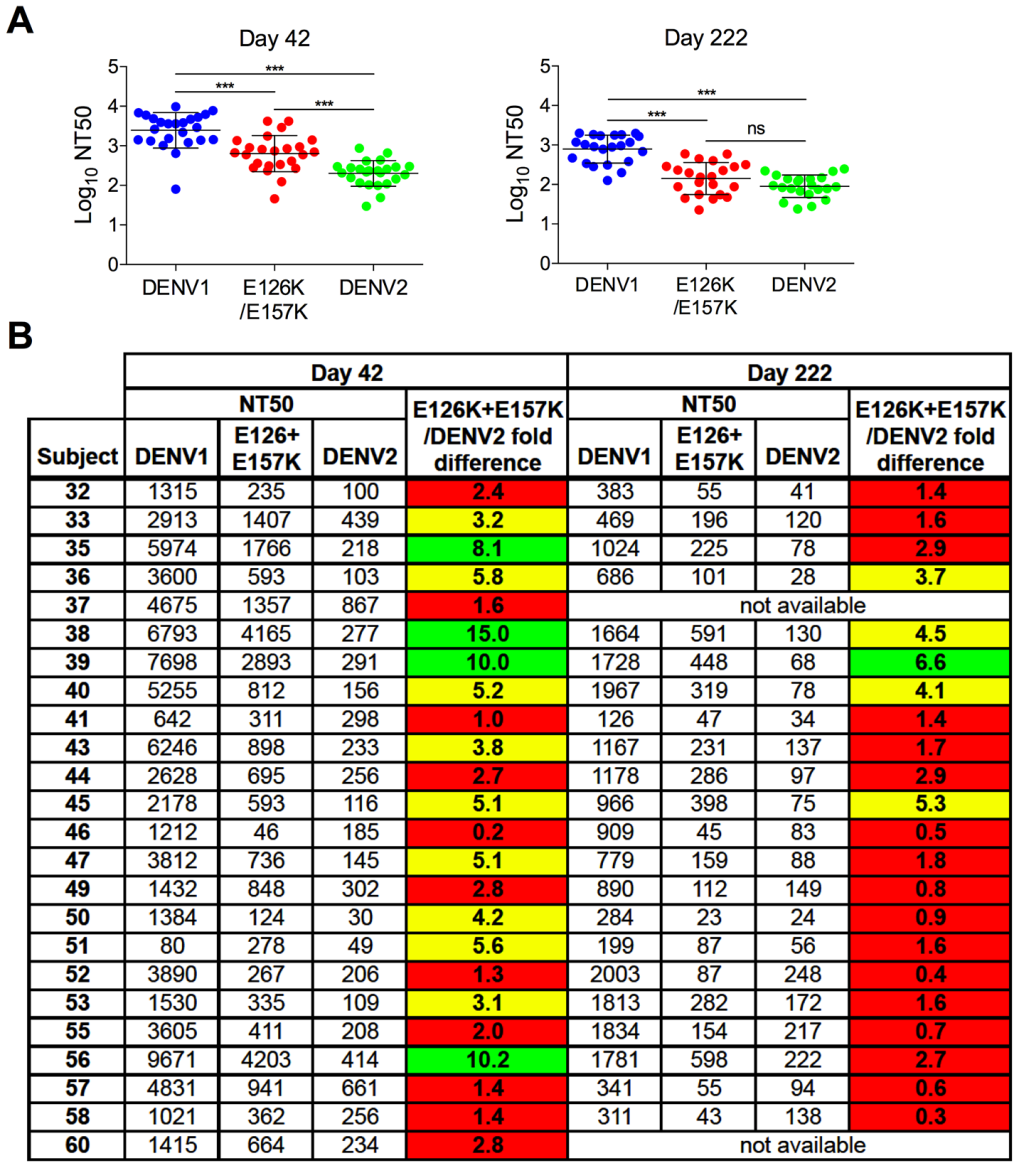
Cross-sectional analysis of the contribution of the E126K/E157K epitopes on TS-neutralization. Sera from an additional 21 DENV1 vaccine recipients collected on days 42 and 222 (when available) were tested for their capacity to neutralize WT DENV1, DENV2 and DENV1 variant E126K/E157K RVPs. (**A**) The mean neutralization potency (Log NT50) of the sera against each virus is shown; error bars represent one standard deviation of the mean. ***p<0.0001; ns, p = 0.15. (**B**) The neutralization titers (NT50) of the sera against WT DENV1, DENV2 and DENV1 E126K/E157K RVPs are presented. The fold-difference in sensitivity between DENV1 variant E126K/E157K RVPs and DENV2 RVPs was determined by the equation (NT50 DENV1 E126K/E157K)/(NT50 DENV2). Red shading indicates a <3 fold difference, yellow shading corresponds to a 3–6 fold difference, and green shading reflects a >6 fold difference.

Longitudinal analysis revealed that the sensitivity of the E126K/E157K variant to neutralization by DENV1-immune sera varied with time and among the three subjects tested. For Subject 43, the contribution of antibodies sensitive to mutations at positions E126 and E157 increased with time post-vaccination. By day 222, DENV2 and DENV1 E126K/E157K RVPs were neutralized to an equivalent degree (**Figure 6a and b**). The reduced sensitivity of DENV1 E126K/E157K RVPs to neutralization was observed at earlier time points in the sera of Subject 57, suggesting the TS-antibody response in this individual was focused early on epitopes impacted by mutating these two residues (**Figure 6b**). In contrast, while the sensitivity of DENV1 E126K/E157K RVPs to neutralization was reduced in comparison to WT at all time points sampled from Subject 39 (from 2–6 fold), mutation of these two residues alone did not ablate the entire TS response (**Figure 6b**).

To better understand the contribution of epitopes defined by the E126K and E157K mutations in the TS response to the DENV1Δ30 vaccine in a larger sample set, we assayed sera collected from an additional 21 DENV1 vaccine recipients at two time points post-vaccination (day 42 and 222; day 222 was collected 42 days after the second vaccine dose was administered). Antibody dose-response curves were generated for each sample using WT DENV1, WT DENV2, and DENV1 E126K/E157K RVPs as described above. The NT50 values calculated from this cross-sectional study are presented in **Figure 7a and b**. As expected, vaccine sera most efficiently neutralized DENV1; DENV1 E126K/E157K and DENV2 RVPs were significantly less sensitive to neutralization at both time points studied (p<0.0001 for both comparisons) (**Figure 7a**). While comparisons of the neutralization titer against DENV1 E126K/E157K and DENV2 revealed differences at day 42 post-vaccination (p<0.0001), by day 222 no significant difference was observed (p = 0.15).

On average, we observed a 3.5-fold decrease in neutralization titers measured with DENV1 RVPs between day 42 and day 222 post-vaccination. This decay was more rapid than the change in DENV2-reactive titers measured at these two time points (mean 2.4-fold decrease) (p<0.01). Of interest, the ability of DENV1 sera to neutralize the E126K/E157K variant reactive antibody declined most dramatically between days 42 and 222 (a mean 4.8-fold decrease). This decline in sensitivity to neutralization was more rapid that that observed with either DENV1 (p<0.05) or DENV2 (p<0.0001) RVPs. This data is in agreement with our interpretation that over time, antibodies in DENV1-immune sera become more focused on epitopes containing residues E126 and E157. On average, RVPs containing mutations at these positions become more difficult to neutralize with time post-vaccination.

Analysis of individual neutralization titers revealed the same pattern (**Figure 7b**). On day 42, the serum of roughly half the recipients neutralized DENV1 E126K/E157K RVPs less efficiently than WT DENV1 RVPs. The contribution of antibodies binding epitopes impacted by mutation of E126 and E157 was monitored as the difference in neutralization sensitivity of the DENV1 E126K/E157K mutant and DENV2 RVPs (highlighted in red, yellow, and green to indicate 0–3, 3–6, and >6 fold differences in NT50). In 46% of the day 42 samples tested, the neutralization titer of the E126K/E157K variant was found to be within 3-fold of the cross-reactive DENV2 neutralization titer (**Figure 7b**). By day 222 post-vaccination, 77% of the NT50 values obtained with the E126K/E157K variant were within 3-fold of the DENV2 neutralization titer, confirming the trend observed in the longitudinal analysis described above. Together, these data identify a significant functional contribution of epitopes containing residues E126 and E157 in the TS DENV1 response that increases with time.

## Discussion

Interpreting the serology of flaviviruses is hindered by extensive cross-reactivity and significant differences in the functional properties of antibodies that bind different epitopes on the virion [23], [35], [36], [39]–[42]. Several approaches have been employed to deconstruct the complexity of the polyclonal antibody response to DENV. Biochemical studies using recombinant proteins or subviral particles have identified mutations in the E protein that reduce recognition by antibodies in DENV immune sera in an ELISA or Western blot assay format [23], [87], [88]. A limitation of this type of approach is that it does not account for the marked difference in neutralization potential among antibodies of varying specificity. A neutralization response driven by the contribution of a low concentration of potent neutralizing antibodies may be difficult to detect using biochemical studies.

The antibody repertoire elicited by DENV infection or vaccination has also been investigated using approaches that allow study of the functional properties of antibodies in immune sera. Exciting advances have been made towards understanding the DENV antibody repertoire through the analysis of the specificity of human mAbs [39], [49], [51]. A strength of this approach is that it enables functional analysis of antibody specificities that make up the polyclonal immune response. While this approach reveals the specificity of DENV-reactive memory B-lymphocytes, it is unknown how faithfully these methods capture the breadth of the polyclonal humoral immune response *in vivo*, and how screening bias impacts the mAbs selected for study. A recent study of 26 DENV-reactive human mAbs obtained from subjects immunized with the rDEN1Δ30 vaccine candidate studied herein identified only three mAbs that neutralize DENV [50]. The functional specificity of the polyclonal DENV immune response has also been investigated by depletion of immune sera using recombinant proteins. These studies suggested antibodies that bind E-DIII are not a significant component of the neutralizing antibody response and play only a modest role in protection in the AG129 mouse model of DENV infection/disease [31], [89]. This finding was confirmed using infectious clones encoding mutations in E-DIII [90].

The number of epitopes that contribute to the neutralizing activity of the polyclonal antibody response to DENV remains unknown. To date, insight into where neutralizing antibodies present in human sera bind DENV has been limited principally to negative data. The goal of the present study was to identify the epitopes recognized by neutralizing antibodies of the type-specific DENV1 response following vaccination. We undertook a mutagenesis approach to identify amino acid substitutions that conferred a reduction in sensitivity to neutralization by DENV1 immune sera, but not cross-reactive antibodies present in DENV2 sera. We created a library of DENV1 RVPs encoding mutations at residues predicted to be surface-accessible on the mature virion that differ between DENV1 and DENV2. We identified single amino acids in E-DI (E126) and E-DII (E157) that when mutated together abrogated a large portion of the TS-neutralizing antibody response in 77% of vaccinated subjects.

That two amino acid substitutions were sufficient to significantly reduce the TS-antibody response in the majority of vaccinees was a surprise. These data suggest the TS-antibody response is remarkably focused. Similar conclusions were reached in studies of the complexity of the polyclonal antibody response to HIV-1 and influenza [91]–[94]. Residues E126 and E157 are very conserved among DENV1 viruses; the mutations identified in our study were found only once (E126K) or not at all (E157K) among 1,398 DENV1 sequences available for *in silico* analysis [95]. Thus, the ability of DENV1 to escape from neutralization by mutation may be limited by the functional pressure of cross-reactive antibody and a substantial fitness cost. A more detailed analysis of the functional consequences of mutations at these residues is underway.

While the TS-immune response of a majority of volunteers in our study was focused significantly on epitopes affected by mutations at E126 and E157, these changes had a somewhat reduced impact on the potency of immune sera from five volunteers. This suggests that additional residues are involved in the fine specificity of the response and will require further study. Limited secondary screening with a subset of the panel of DENV1 variants identified residue 203 as a significant contributor to TS-neutralization patterns of Subject 38 (Figure S3), but not the others. As this residue is located within 13 Å from residue 126, it is possible that mutations at this position impact recognition of the same or overlapping epitopes in E-DII.

While, to our knowledge, residues 126 and 157 on DENV1 E protein have not yet been identified in neutralization escape studies with DENV mAbs, recent studies have identified a complex epitope in proximity to E157. Studies of the human anti-WNV mAb CR4354 identified an epitope at the junction of E-DI and E-DII that exists only on the intact virion, and not on soluble E protein [96]. Four human mAbs have since been characterized that bind in the same region. The footprint of the TS DENV1 mAb DENV HM14c10 has been solved using cryo-electron microscopy reconstruction and has confirmed the discontinuous structure of the epitope and its similarity to CR4354 [47], [52]. Neutralization escape mutants have been identified for HM14c10 as well as three additional human mAbs thought to recognize quaternary epitopes on DENV; each escape variant was mutated around the DI–II hinge region and within the CR4354 footprint [47], [48]. The E157K mutation identified in our study is close to the hinge region. However, it remains unclear if antibodies that bind DENV1 at this location engage a quaternary epitope similar to CR4354, as this residue falls just outside the DI–DII hinge epitopes defined by the footprints of WNV CR4354 or DENV HM14c10. Furthermore, the 12 surface-exposed residues that differ between DENV1 and DENV2 present in the published DI–DII hinge epitope footprints did not themselves markedly reduce sensitivity to neutralization by DENV1 sera in this screen.

The dense arrangement of E proteins on the virion complicates our understanding of antibody recognition. Many well-characterized epitopes are not predicted to be accessible for antibody binding using existing static models of the mature DENV structure [23], [76], [97]. For example, antibodies that bind the E-DII fusion loop are a significant component of the CR repertoire produced by mouse and human following flavivirus infection, yet bind an epitope not predicted to be accessible for antibody recognition [23]. The accessibility of cryptic epitopes like the DII-FL has been shown to be governed by several factors [74], [81]. Viruses exist as an ensemble of structures at equilibrium (reviewed by [84], [98]). “Breathing” of envelope proteins incorporated into the flavivirus virion has been shown to alter epitope accessibility and sensitivity to neutralization by monoclonal and polyclonal antibody [81], [82], [99]. In addition, many antibodies are sensitive to the maturation state of the virus particle [74]. Engineered mutations or naturally occurring variation have the potential to alter the heterogeneity or dynamics of the virion, and therefore may impact epitope exposure. While we have identified amino acids that contribute to the type-specific recognition of the DENV1 Western Pacific strain, epitopes containing these amino acids may play a reduced or enhanced role in the context of other viral E protein sequences. Of interest, ∼30% of the vaccine recipients studied within neutralize a related DENV1 strain 16007 more efficiently than the WP strain used in the DENV1 vaccine candidate (data not shown). This enhanced recognition may reflect differences in the “breathing” of these two viruses that alter the accessibility of epitopes on the virus particle, as suggested in a recent study [100]. As our analysis was focused on surface accessible differences between the DENV1 and DENV2 components of the vaccine, it is possible that amino acid variation among DENV1 strains and differences in the extent of “viral breathing” (and therefore epitope exposure) will impact the pattern of type-specific recognition of different DENV1 strains. It remains to be determined whether the two resides characterized within will be useful as a generalizable signature of a type-specific humoral response for all DENV1 viruses. Evaluating the contribution of this structural complexity towards neutralization sensitivity is a critical component of understanding the antigenic surface of flaviviruses and how this varies among strains within and between serotypes.

The identification of major targets of the TS-neutralizing antibody response to DENV1 vaccination represents an important step toward a more complete understanding of the humoral immune response to DENV. Whether epitopes including residues E126 and E157 contribute significantly to the neutralizing antibody response to natural infection or vaccination with all four DENV serotypes remains to be determined. The four serotypes of DENV share a 63% or greater amino acid identity and presumably the same overall virion structure. The epitopes engaged by TS-neutralizing antibodies elicited by infection of other DENV serotypes are unknown. Of note, the introduction of the reciprocal mutations at positions K126 and K157 of DENV2 NGC did not markedly shift the neutralization curve of DENV2 immune sera obtained from vaccine recipients (Figure S4). These data suggest that antibodies that contribute to the TS-neutralizing antibody response may bind distinct epitopes on all four DENV serotypes. Beyond the identification of significant epitopes in DENV, the data and experimental approaches described within provide insight into mutagenesis approaches for deconstructing the functional components of the polyclonal DENV antibody response and will be used to guide future studies on the functionally important epitopes involved in the neutralization of the other three DENV serotypes.

## Materials and Methods

### Cell lines

All cell lines were maintained at 37**°**C in the presence of 7% CO_2_. HEK-293T cells were passaged in complete Dulbecco's modified Eagle medium (DMEM) containing Glutamax (Invitrogen, Carlsbad, CA), supplemented with 7.5% fetal bovine serum (FBS) (HyClone, Logan, UT) and 100 U/ml penicillin-streptomycin (PS) (Invitrogen, Carlsbad, CA). Raji-DCSIGNR cells were passaged in RPMI-1640 medium containing Glutamax (Invitrogen, Carlsbad, CA), supplemented with 7.5% FBS and 100 U/ml PS.

### DENV immune sera and antibodies

Sera from recipients of phase I studies of candidate monovalent DENV1 or DENV2 vaccines were obtained for study. Initial screening studies were performed using sera pooled from two or three recipients of the DENV1 and DENV2 vaccines, respectively, collected 2–3 years post-vaccination. Neutralizing antibody responses from 24 participants of a DENV1 vaccine study were studied individually [86]; sera were collected for study on the indicated day post-vaccination.

### Ethics statement

Clinical studies were conducted at the Center for Immunization at the Johns Hopkins Bloomberg School of Public Health under an investigational new drug application reviewed by the United States Food and Drug Administration. The clinical protocol and consent form were reviewed and approved by the NIAID Regulatory Compliance and Human Subjects Protection Branch, the NIAID Data Safety Monitoring Board, the Western Institutional Review Board, and the Johns Hopkins University Institutional Biosafety Committee (ClinicalTrials.gov identifiers; NCT00473135, NCT00920517). Written informed consent was obtained from each participant in accordance with the Code of Federal Regulations (21 CFR 50) and International Conference on Harmonisation guidelines for Good Clinical Practice (ICH E6).

### Plasmids

Plasmids encoding a WNV sub-genomic replicon and DENV1 WP CprME structural genes have been described previously [77], [81], [101]. An expression construct of the CprME gene of the DENV2 NGC strain was constructed using similar methods and will be described elsewhere (VanBlargan and Pierson, unpublished data). Plasmids encoding structural gene variants with up to three amino acid substitutions were produced by site-directed mutagenesis using the Quikchange Mutagenesis kit (Stratagene, La Jolla, CA) according to the manufacturer's instructions. All plasmids used in this study were propagated in Stbl2 bacteria grown at 30**°**C (Invitrogen, Carlsbad, CA).

### Selection of DENV1 residues for mutagenesis

To identify amino acids recognized by TS-neutralizing antibodies in vaccine sera, we targeted residues for mutagenesis that differed between the DENV1 and DENV2 strains used in the NIAID tetravalent vaccine candidate. The envelope proteins of the DENV1 WP strain and the DENV2 NGC strain differ by 158 amino acids. As a metric to narrow our mutagenesis efforts, we focused on residues predicted to be exposed on the surface of the virion. Surface accessibility was estimated using solvent accessible surface areas of the residues determined from the crystal structure of the E protein dimer (PDB ID: 10AN), with a cut-off value of 30 Å^2^ (UCSF Chimera package) [102], [103]. Residues were then further restricted by modeling the dimer onto the mature virion (PDB ID: 1THD) [71]. Residues exposed on the surface of the virion were then selected, narrowing the list of candidates to 68 [71]. Admittedly, because our selection scheme was based on a static model of the mature virion, this minimalist approach to selecting a core panel of residues had the potential to be complicated by the structural heterogeneity and dynamics of the virus particle. Virion structure is influenced by the maturation state and structural dynamics of the virion [15], [74], [81]. Both have the potential to increase the number of residues that may contribute to TS-antibody recognition. Additionally, structural studies of DENV2 at 37**°**C revealed the virus not only becomes considerably more heterogeneous at this temperature but also appears to adopt a distinct structure(s) at this temperature [104], [105].

### Production of DENV RVPs

DENV RVPs were produced by complementation of a WNV replicon with plasmids encoding the structural genes of DENV as described previously [81], [101]. Briefly, pre-plated HEK-293T cells were transfected with plasmids encoding the WNIIrepG/Z replicon and DENV CprME in a 1∶3 ratio by mass, using Lipofectamine LTX (Invitrogen, Carlsbad, CA) in accordance with the manufacturer's instructions. Four hours post-transfection, culture media were replaced with a low-glucose formulation of DMEM containing 25 mM HEPES (Invitrogen, Carlsbad, CA), 7% FBS, and 100 U/ml PS, and incubated at 30**°**C. RVP-containing supernatant was collected at multiple time points starting at 72 hours post-transfection, filtered using a 0.22 µm syringe filter, and stored at −80**°**C. The low-glucose DMEM was replenished after each harvest.

To generate more homogenous populations of RVPs with low to undetectable uncleaved prM, complementation experiments were modified to include a plasmid expressing human furin (furin DENV) in a 1∶3∶1 ratio of replicon, CprME, and furin by mass. In order to generate RVP populations that retained significant levels of uncleaved prM (FI-DENV), medium from transfected cells was exchanged with medium supplemented with 50 µM furin inhibitor (FI) Dec-RVKR-CMK at four hours post-transfection (Enzo Life Sciences, Farmingdale, NY).

The efficiency of prM cleavage in RVP preparations was determined by Western blotting as previously described [74], [106]. Briefly, DENV RVPs were partially purified over a 20% sucrose cushion by ultra-centrifugation. Pelleted RVPs were lysed with in buffer containing 1% Trition, 100 mM Tris, 2 M NaCl, and 100 mM EDTA. The protein content of lysates was analyzed using E- and prM-reactive mAbs (4G2 and GTX128093 (Genetex), respectively) at 1 µg/ml. The efficiency of prM cleavage was evaluated on blots normalized by loading equivalent E protein.

### Measuring the infectious titer of DENV RVPs

The infectious titer of each RVP stock was determined using Raji cells that express the flavivirus attachment factor DCSIGNR as described previously [78], [101], [107]. Cells were infected with serial two-fold dilutions of transfection supernatant, incubated at 37°C for two days, and then scored for GFP expression by flow cytometry. Only data from the linear portion of the virus dose-infectivity curves were used to compare RVP titers. Infectious titer was calculated using the formula: IU/sample volume = (percent GFP positive cells)×(number of cells)×(dilution factor).

### DENV neutralization assays

DENV RVP stocks were diluted and incubated with serial dilutions of mAb or serum for one hour at room temperature prior to the addition of Raji-DCSIGNR cells, unless specified otherwise. Infections were carried out at 37**°**C for 48 hours and infectivity was scored as the fraction of GFP-expressing cells determined using flow cytometry. Antibody-dose response curves were analyzed using non-linear regression analysis (with a variable slope) (Graphpad Software, La Jolla, CA). Data are expressed as the concentration of antibody (EC50) or serum dilution (NT50) required to reduce infection by half.

### Statistical analysis

Statistical analyses were performed using Prism software (GraphPad). Log EC50 or Log NT50 values were compared using Student's *t*-test when comparing two samples. For comparisons of more than two samples, Log NT50 values were compared by one-way ANOVA followed by Tukey's multiple comparisons test or, where indicated, Šidák correction for multiple comparisons.

## Supporting Information

Figure S1 **Sensitivity of DENV1 variants to neutralization by mAb E60.** Each member of the panel of 54 DENV1 variants was tested in parallel with WT DENV1 for sensitivity to neutralization by CR mAb E60. Dose response curves were generated by incubating serial dilutions of antibody with RVPs for one hour at room temperature, before addition of Raji-DCSIGNR cells. EC50 values for each curve were determined by nonlinear regression analysis using Prism software (GraphPad), and are depicted as the fold-change in neutralization sensitivity from WT DENV1 ([EC50 WT]/[EC50 variant]). The experiment was repeated for a subset of the panel to capture the variability of the assay; error bars, when present, represent standard error of the mean from 2–3 independent experiments for 25 of the variants.
(TIFF)

Figure S2 **Neutralization of DENV1 E126K/E157K by DENV1 mAbs.** DENV1 E126K/E157K RVPs were tested in parallel with WT DENV1 for sensitivity to neutralization by a panel of six DENV1 mAbs that bind diverse epitopes on DIII [35]. The mAbs used were (**A**) E90 (N-terminal region and BC-loop); (**B**) E98 (F- and G-strands); (**C**) E99 (A-strand); (**D**) E100 (A-strand, BC and DE loops); (**E**) E102 (N-terminal region and the BC loop); and (**F**) E106 (A-strand, BC, DE, and FG loops). Dose response curves shown are representative of two independent experiments; error bars represent the standard error of duplicate infections. EC50 values for WT and the variant were less than 2-fold different in all cases.
(TIFF)

Figure S3 **Neutralization of additional DENV1 variants by sera from DENV1 vaccine recipients.** While the TS-immune response of a majority of volunteers in our study was focused significantly on epitopes affected by mutations at E126 and E157, these changes had a reduced impact on the potency of immune sera from five volunteers (Subjects 36, 38, 39, 40, and 45). Secondary screening of day 222 sera from these subjects was performed with a panel of ten of DENV1 variants shown to modestly decrease the potency of the DENV1 pooled serum (**Figure 3**). Only a role for mutant K203N in modulating the neutralization sensitivity of DENV1 immune sera of Subject 38 was identified as significant using our screening metric (<3-fold difference in NT50 between variant K203N and DENV2, n = 2). Antibody-dose response curves from a representative screening study are displayed.
(TIFF)

Figure S4 **Effect of mutations at residues 126 and 157 on DENV2 RVPs.** To test whether the residues 126 and 157 are targets of TS antibodies in DENV2 sera, a DENV2 NGC variant was constructed containing the reciprocal mutations, K126E and K157E. (**A**) DENV2 K126E/K157E RVPs were tested for sensitivity to neutralization by pooled DENV2 sera. Representative dose-response curves are shown on the left; error bars represent the standard error of duplicate infections. NT50 values from four independent experiments are shown on the right and reveal a modest 1.5-fold increase in neutralization sensitivity of the variant (p<0.05). (**B**) DENV2 K126E/K157E was tested for sensitivity to neutralization by CR mAb E60. Representative dose-response curves are shown on the left; error bars represent the standard error of duplicate infections. NT50 values from four independent experiments are shown on the right, and reveal a similar 1.4-fold increase in sensitivity to neutralization compared to WT DENV2, though this difference did not reach statistical significance (p = 0.11).
(TIFF)
