# Supplementary figures and images for: The Type-Specific Neutralizing Antibody Response Elicited by a Dengue Vaccine Candidate Is Focused on Two Amino Acids of the Envelope Protein
Source: PLoS Pathog. 2013 Dec 5;9(12):e1003761. doi: 10.1371/journal.ppat.1003761 (PMC3857832; doi:10.1371/journal.ppat.1003761)

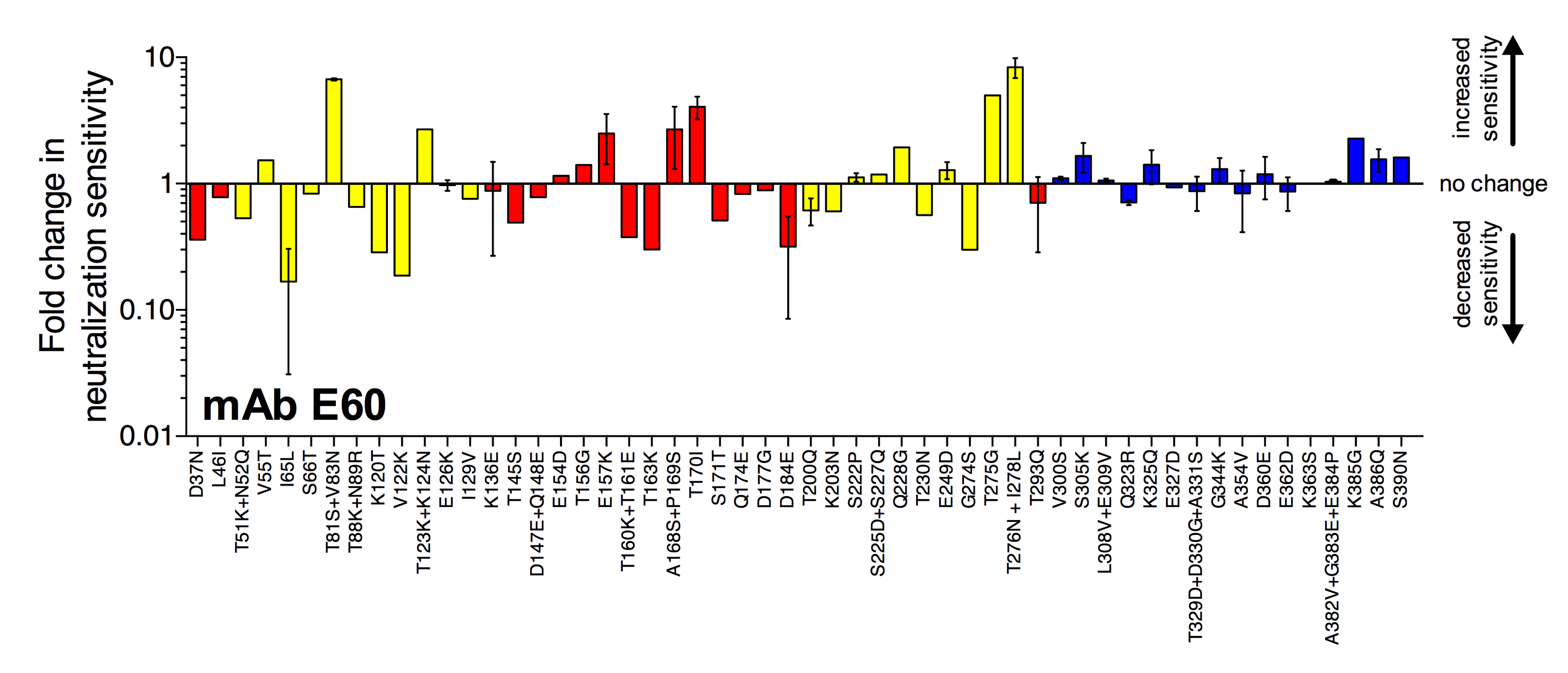

Supplement: Figure S1 — Sensitivity of DENV1 variants to neutralization by mAb E60. Each member of the panel of 54 DENV1 variants was tested in parallel with WT DENV1 for sensitivity to neutralization by CR mAb E60. Dose response curves were generated by incubating serial dilutions of antibody with RVPs for one hour at room temperature, before addition of Raji-DCSIGNR cells. EC50 values for each curve were determined by nonlinear regression analysis using Prism software (GraphPad), and are depicted as the fold-change in neutralization sensitivity from WT DENV1 ([EC50 WT]/[EC50 variant]). The experiment was repeated for a subset of the panel to capture the variability of the assay; error bars, when present, represent standard error of the mean from 2–3 independent experiments for 25 of the variants. (TIFF) [file ppat.1003761.s001.tiff]

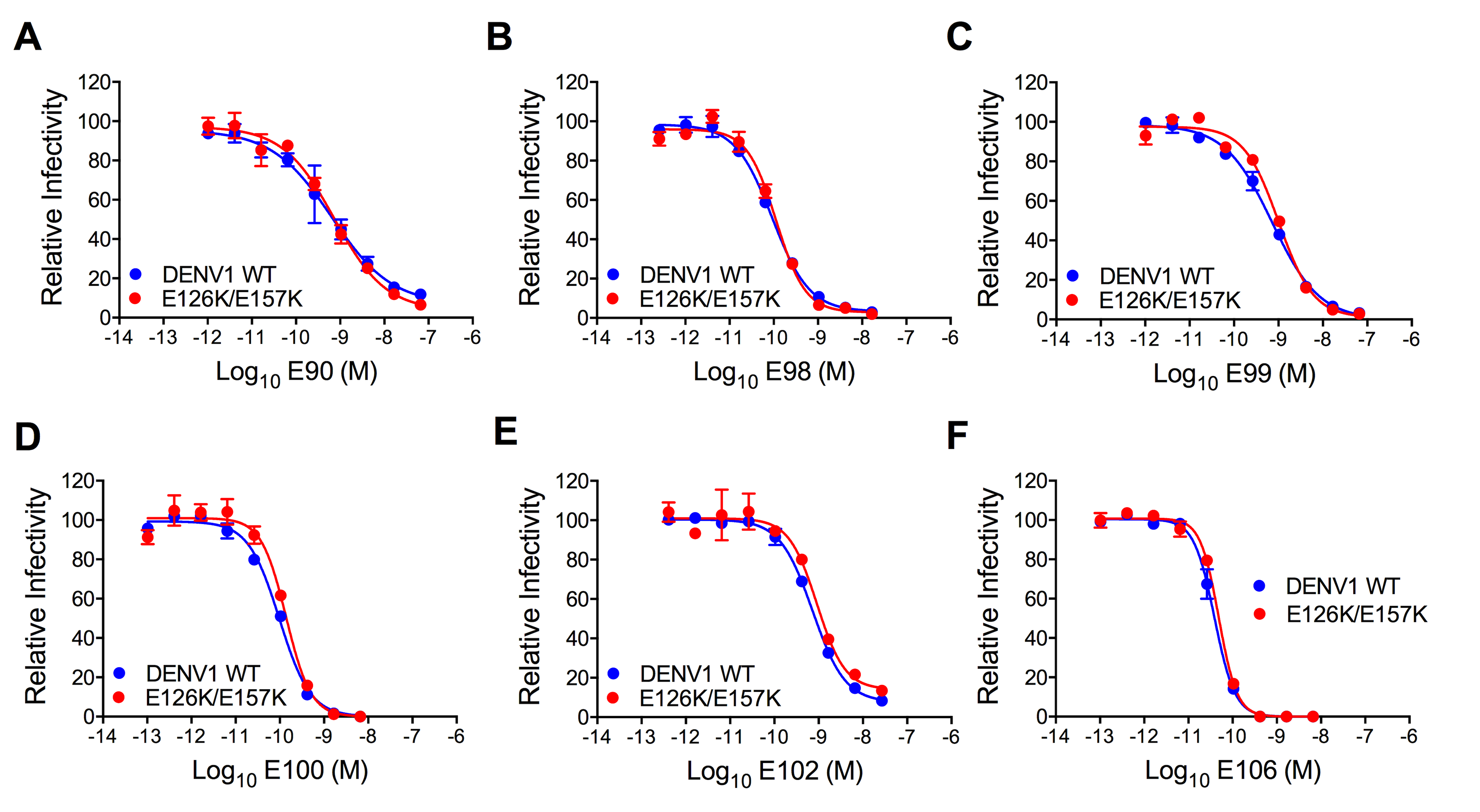

Supplement: Figure S2 — Neutralization of DENV1 E126K/E157K by DENV1 mAbs. DENV1 E126K/E157K RVPs were tested in parallel with WT DENV1 for sensitivity to neutralization by a panel of six DENV1 mAbs that bind diverse epitopes on DIII [35]. The mAbs used were (A) E90 (N-terminal region and BC-loop); (B) E98 (F- and G-strands); (C) E99 (A-strand); (D) E100 (A-strand, BC and DE loops); (E) E102 (N-terminal region and the BC loop); and (F) E106 (A-strand, BC, DE, and FG loops). Dose response curves shown are representative of two independent experiments; error bars represent the standard error of duplicate infections. EC50 values for WT and the variant were less than 2-fold different in all cases. (TIFF) [file ppat.1003761.s002.tiff]

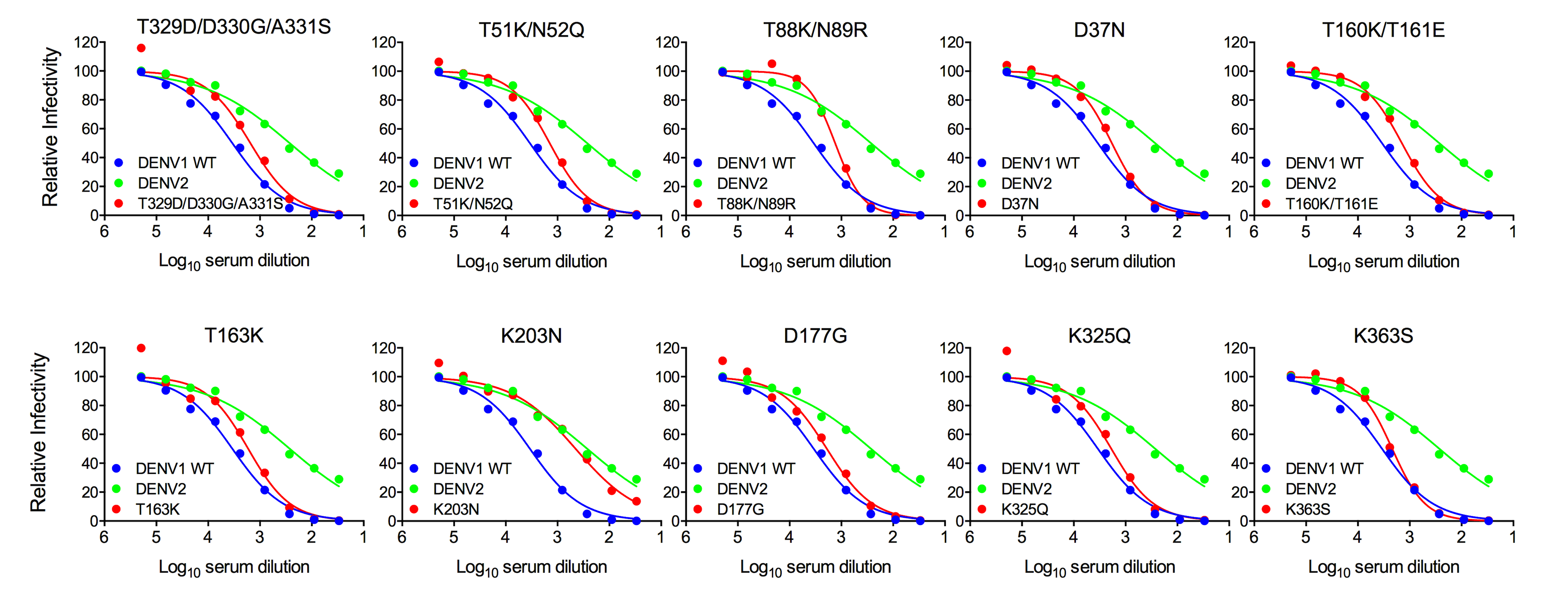

Supplement: Figure S3 — Neutralization of additional DENV1 variants by sera from DENV1 vaccine recipients. While the TS-immune response of a majority of volunteers in our study was focused significantly on epitopes affected by mutations at E126 and E157, these changes had a reduced impact on the potency of immune sera from five volunteers (Subjects 36, 38, 39, 40, and 45). Secondary screening of day 222 sera from these subjects was performed with a panel of ten of DENV1 variants shown to modestly decrease the potency of the DENV1 pooled serum ( Figure 3 ). Only a role for mutant K203N in modulating the neutralization sensitivity of DENV1 immune sera of Subject 38 was identified as significant using our screening metric (<3-fold difference in NT50 between variant K203N and DENV2, n = 2). Antibody-dose response curves from a representative screening study are displayed. (TIFF) [file ppat.1003761.s003.tiff]

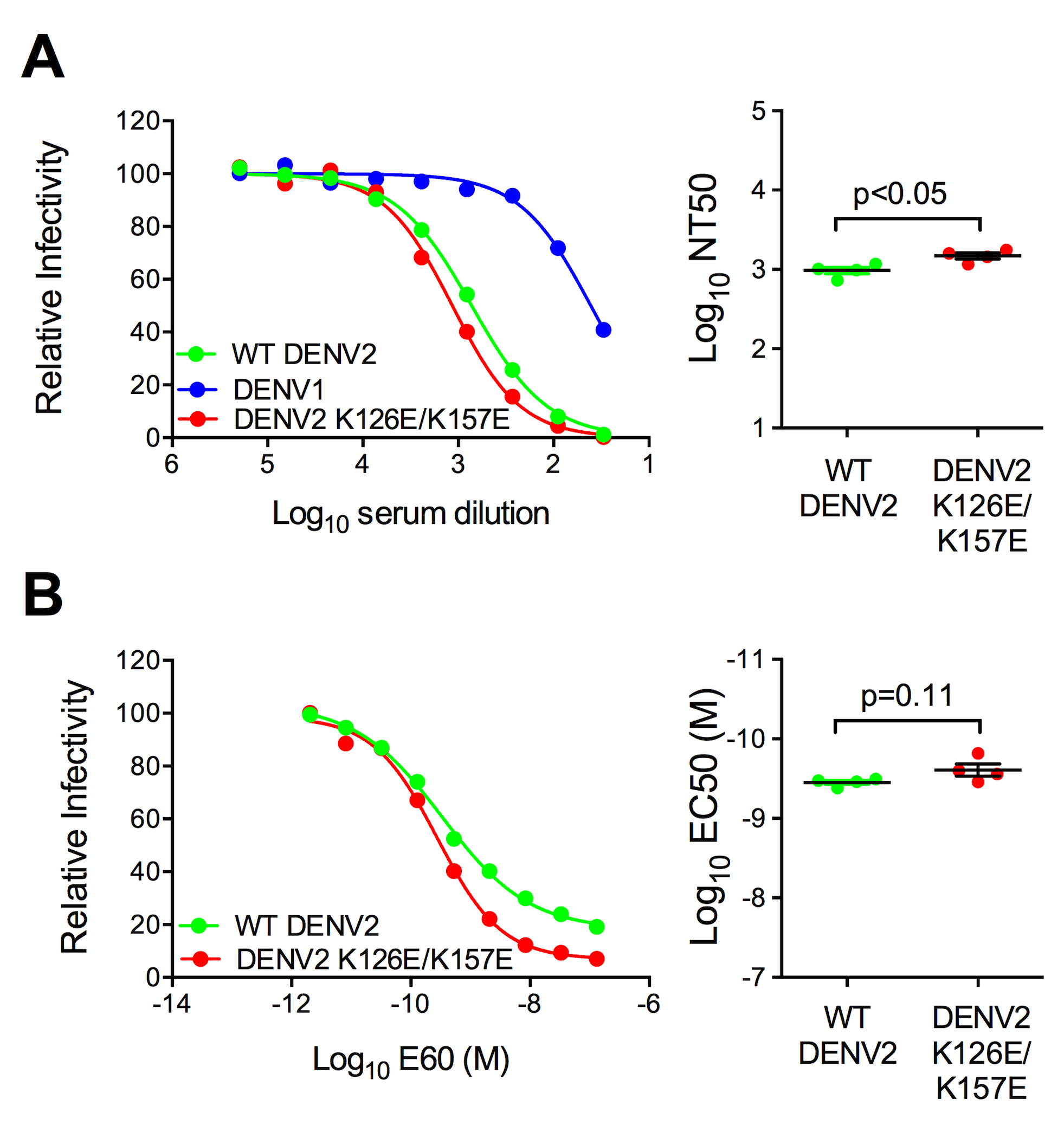

Supplement: Figure S4 — Effect of mutations at residues 126 and 157 on DENV2 RVPs. To test whether the residues 126 and 157 are targets of TS antibodies in DENV2 sera, a DENV2 NGC variant was constructed containing the reciprocal mutations, K126E and K157E. (A) DENV2 K126E/K157E RVPs were tested for sensitivity to neutralization by pooled DENV2 sera. Representative dose-response curves are shown on the left; error bars represent the standard error of duplicate infections. NT50 values from four independent experiments are shown on the right and reveal a modest 1.5-fold increase in neutralization sensitivity of the variant (p<0.05). (B) DENV2 K126E/K157E was tested for sensitivity to neutralization by CR mAb E60. Representative dose-response curves are shown on the left; error bars represent the standard error of duplicate infections. NT50 values from four independent experiments are shown on the right, and reveal a similar 1.4-fold increase in sensitivity to neutralization compared to WT DENV2, though this difference did not reach statistical significance (p = 0.11). (TIFF) [file ppat.1003761.s004.tiff]
